# Manufacture of High-Efficiency and Stable Lead-Free Solar Cells through Antisolvent Quenching Engineering

**DOI:** 10.3390/nano12172901

**Published:** 2022-08-23

**Authors:** Amal Bouich, Julia Marí-Guaita, Bernabé Marí Soucase, Pablo Palacios

**Affiliations:** 1 Escuela Técnica Superior de Ingeniería del Diseño, Universitat Politècnica de València, 46022 València, Spain; 2 Instituto de Energía Solar, ETSI Telecomunicación, Universidad Politécnica de Madrid, Ciudad Universitaria, s/n, 28040 Madrid, Spain; 3 Departamento de Física Aplicada a las Ingenierías Aeronáutica y Naval, ETSI Aeronáutica y del Espacio, Universidad Politécnica de Madrid, Pz. Cardenal Cisneros, 3, 28040 Madrid, Spain

**Keywords:** perovskite solar cell, MASnI_3_, antisolvent treatment, photoconversion efficiency, photochemical stability

## Abstract

Antisolvent quenching has shown to significantly enhance several perovskite films used in solar cells; however, no studies have been conducted on its impact on MASnI_3_. Here, we investigated the role that different antisolvents, i.e., diethyl ether, toluene, and chlorobenzene, have on the growth of MASnI_3_ films. The crystallinity, morphology, topography, and optical properties of the obtained thin films were characterized by X-ray diffraction (XRD), scanning electron microscopy (SEM), photoluminescence (PL) measurements, and UV–visible spectroscopy. The impact of the different antisolvent treatments was evaluated based on the surface homogeneity as well as the structure of the MASnI_3_ thin films. In addition, thermal annealing was optimized to control the crystallization process. The applied antisolvent was modified to better manage the supersaturation process. The obtained results support the use of chlorobenzene and toluene to reduce pinholes and increase the grain size. Toluene was found to further improve the morphology and stability of thin films, as it showed less degradation after four weeks under dark with 60% humidity. Furthermore, we performed a simulation using SCAPS-1D software to observe the effect of these antisolvents on the performance of MASnI_3_-based solar cells. We also produced the device FTO/TiO_2_/MASnI_3_/Spiro-OMeTAD/Au, obtaining a remarkable photoconversion efficiency (PCE) improvement of 5.11% when using the MASnI_3_ device treated with chlorobenzene. A PCE improvement of 9.44% was obtained for the MASnI_3_ device treated with toluene, which also showed better stability. Our results support antisolvent quenching as a reproducible method to improve perovskite devices under ambient conditions.

## 1. Introduction

Solar energy is considered a basis to obtain clean and abundant energy; therefore it has become essential to take advantage of solar radiation as an energy source in the field of photovoltaics [1]. Recently, organic and inorganic perovskites, especially those based on methylammonium halides, have become attractive and promising research materials. Perovskite solar cells (PSC) showing power conversion efficiencies higher than 23% have been reported [2]. Perovskite materials also have remarkable optical and electronic properties, as they allow light to be absorbed in a wide wavelength for a long time, charge carriers to have a long diffusion length, and excitons to have small binding energy, which produces electrons and holes [3,4,5]. Furthermore, for perovskite thin film fabrication, a variety of techniques have been used, including one-step and two steps deposition [6], evaporation [7], and thermal deposition [8]. One-step deposition carried out via spin coating demonstrated an excellent capability to fabricate PSCs. Moreover, the PSCs showed homogeneous and compact thin films when fabricated using the spin coating technique. It has been shown that the morphology of perovskite thin films plays a vital role in device performance. Many methods and treatments have been introduced to optimize the product quality by improving the surface homogeneity and crystallinity of the films. To achieve a good crystallization of thin films, several strategies are used, such as evaporation, cooling, heating, addition of an antisolvent—also in combination—so to slow the solubility in a saturated solution. The film surface’s supersaturation level and thermal annealing were investigated by adding different antisolvents. Several physicochemical properties affect this process, resulting in complex exchanges occurring simultaneously. Improving the performance of solar cells requires a thorough understanding of the mechanisms involved and the proper use of adequate antisolvent treatments [9,10].

A PSC with methylammonium lead iodide (MAPbI_3_) as an absorber has the highest conversion efficiency, but lead (Pb) contained in the material may have dramatic health consequences; therefore, it is highly appropriate and necessary to explore the possibility of substituting tin (Sn) for lead in MAPbI_3_ and improve the efficiency and stability of MASnI_3_-based solar cells. Investigations should start by analyzing the growth of the absorber layer to achieve high crystallinity and optimal morphology, as these features are essential for improving the optical properties of the absorber. Recently, the use of tin halide perovskites as absorbers has been investigated. MASnI_3_-based solar cells are considered good candidates for perovskite solar cells. It has been demonstrated that MASnI_3_ PSC have a power conversion of 6.4% under one sun illumination with an open circuit voltage of 0.88 V. However, their performance varies widely because of an uncontrollable doping effect caused by the introduction of Sn^4+^ through the oxidation of Sn^2+^. Suppressing Sn^2+^ oxidation is, therefore, an appropriate approach to improving and stabilizing the device performance; this can be achieved by adding SnF_2_ or excess SnI_2_, Furthermore, the fabrication process greatly affects the morphology of the perovskite layer. Since tin perovskite crystallizes rapidly and is very soluble, fabrication methods are limited for tin perovskite films [11,12,13,14].

In this work, MASnI_3_ films were treated with different antisolvents to see how they affected the supersaturation of the solvent. We report that complex interactions between the solvent and the antisolvent are related to the film’s physicochemical properties; therefore, choosing the antisolvent type is critical to improve the perovskite film’s morphology and, in turn, boost the PSC performance. The antisolvent effects manifested in the variation of morphology, structure, and composition of the thin films. Toluene (TOL), chlorobenzene (CBZ), and diethyl ether (ET) were the antisolvents used in this investigation. The obtained MASnI_3_ samples were further analyzed by other characterization techniques such as XRD, SEM, and optical and PL analyses. Additionally, we produced the device FTO/TiO_2_/MASnI_3_/Spiro-OMeTAD/Au, which demonstrated an amazing PCE.

## 2. Experimental Procedure

### 2.1. Materials

Tin (II) iodide (SnI_2_) was purchased from Alfa Aesar, Haverhill, MA, USA, methylammonium iodide (MAI) with the formula CH_6_NI was purchased from Tokyo Chemical Industry, Oxford, UK. *N*,*N*-dimethylformamide (DMF) and dimethyl sulfoxide (DMSO) were purchased from Sigma Aldrich, Madrid, Spain, and used for manufacturing the optoelectronic devices. The volume of DMF used was 1ml, while that of DMOS was 95 μL. Diethyl ether anhydrous (ET), chlorobenzene (CBZ), and toluene (Tol) were used as antisolvents, were all purchased from Sigma Aldrich Madrid Spain, and were used as received. Fluorine tin oxide-coated glass (FTO) was used as a substrate with Sheet Resistance of 6–9 Ω/square and Roughness of 34.8 nm.

### 2.2. Film Preparation

The FTO substrates were washed for 15 min in soap, ethanol, acetone, and isopropanol. Compressed air was used to dry the FTO glass. Afterward, the FTO substrates were put in a UV–ozone cleaner for 15 min to eliminate contaminations and wetness on the surface. The FTO substrates were then placed under an inert atmosphere of argon, together with MASnI_3_ samples until characterization could be undertaken. For the elaboration of the films, a 1 M solution of MASnI_3_ was prepared by dissolving equimolar ratios of MAI and SnI_2_ in 1 mL of DMF and 95 μL DMSO at 60 °C for 2 h. When the solution was ready, 50 µL of MASnI_3_ solution was statically spin-coated at 2000 rpm for 20 s, and 100 µL of one of the antisolvents was applied after a certain time from the initiation of the spin program, referred to as the optimized dripping time of 12 s. Then, MASnI_3_ samples were annealed for 5 min at 50 °C, and their surface became black. This initial color change was due to the growth of the perovskite structure; MA requires relatively little energy to begin intercalating between SnI_2_. To avoid thermal shocks, the samples were heated slowly to 100 °C and held there for 5 min, removing any remaining solvent. The samples became dark during this stage of annealing, indicating that the perovskite phase had completely formed. Afterwards, the prepared solution of MASnI_3_ was spin-coated onto the FTO substrate at 2000 rpm for 20 s. Different antisolvents were dripped onto the MASnI_3_ thin films, and subsequently, the as-prepared MASnI_3_ samples were annealed in a vacuum for 20 min at 250 °C. The deposition and annealing processes were carried out in a glove box where all samples were stored until characterization; they were termed fresh samples.

The crystal structure analysis of perovskite thin films was performed using an XRD RIGAKU Ultima IV diffractometer with Cu kα radiation (λ = 1.5418 Å). Surface morphology was investigated through SEM at different magnifications within the Zeiss Auriga Compact, with an applied voltage of 1.5 kV. The film’s topography was characterized using a cross section. The absorption was measured in a UV–visible wavelength range from 300 to 850 nm with an Ocean Optics HR4000 spectrophotometer, and PL was performed using a He–Cd laser driven by a semiconductor laser with a wavelength of λ = 405 nm.

### 2.3. Device Preparation: Gold/Spiro OMeTAD/MASnI_3_/Compact–TiO_2_/FTO/Glass

The MASnI_3_ devices were made onto FTO-coated substrates. Here are the steps involved in the entire process:

(1) The substrates were cut obtaining samples with a size of 2.5 × 2.5 cm^2^.
(2) Zinc powder was sprinkled over the area of the FTO substrate that we wished to etch in a customized holder. This step involved partially etching the FTO substrates where the zinc powder was sprayed with concentrated HCl (2 M).
(3) An ultrasound bath was used to wash the etched substrates with 2% Hellmanex solution for 15 min; then, the substrates were dipped in deionized water. A UV–ozone cleaner was used to treat the etched substrates for 15 min after washing with ethanol, acetone, and isopropanol using the same method and air-drying.
(4) Spray pyrolysis was used to deposit the first TiO_2_ electron blocking layer. TiO_2_ was deposited using titanium diisopropoxide bis (acetylacetonate) diluted in ethanol with oxygen as the carrier gas, by spin coating and heating at 500 °C for 30 min.
(5) Then, the as-prepared solution of MASnI_3_ was spin-coated at 2000 rpm for 20 s and annealed at 100 °C for 5 min on FTO/TiO_2_.
(6) Afterwards, 50 μL of the Spiro-OMetad solution was deposited by spin coating at 4000 rpm for 30 s on top of MASnI_3_/TIO_2_/FTO. The solution was prepared with 28.8 μL of 4-tert-butylpyridine and 17.5 μL of a stock solution of 520 mg mL^−1^ of lithium bis-(tri-fluoromethylsulfonyl) imide in acetonitrile and by dissolving 72.3 mg of (2,2′ 7,7′-tetrakis(*N*,*N*’-di-p-methoxyphenylamine)-9,9′-spirobifluorene) in 1 mL of chlorobenzene.
(7) At the end of the elaboration process of the solar device, a thin layer of gold contact was thermally evaporated on the top of the device.
(8) In order to determine the J–V characteristic curve of the as-deposited photovoltaic devices, we used the Abet Solar simulator with a 1.5 AM filter. A mask was used to define a 0.1 cm^2^ active area for the devices.

### 2.4. Experiment Structure

The samples were prepared, and with Tol, ET, and CBZ were used as antisolvents. The dripping time for all samples was 10 s; 100 µL of each of the different antisolvents was applied. The samples were characterized by XRD, UV–vis spectroscopy, PL, and FESEM. At the end of the experiment, XRD and SEM images were used to determine the stability. During the process of antisolvent selection, all samples were kept in the laboratory for the degradation study at 23 °C and a relative humidity of 60% for 4 weeks. Daily temperature measurements revealed only small fluctuations of 2 °C. Due to heavy precipitation outside, the relative humidity decreased to 53% for a few days during the experiment. All samples were exposed to the same conditions, allowing for a fair comparison between them. To manufacture the Spiro-OMeTAD/MASnI_3_/TiO_2_/FTO device, the TiO_2_ film was prepared outside the glovebox after the perovskite layer and spiro-OMeTAD were deposited inside the glovebox; then, the gold layer was thermally evaporated on the top of the device.

## 3. Results and Discussion

**Figure 1:**
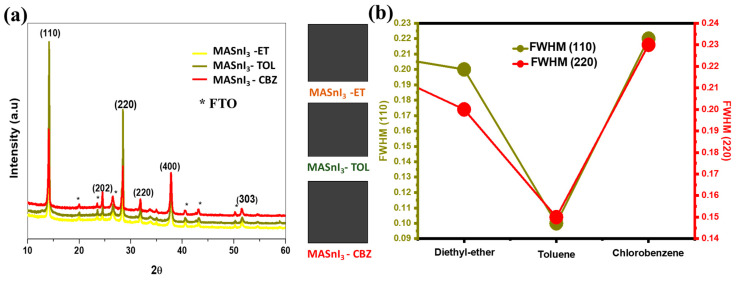
(**a**) XRD patterns of MASnI_3_ films treated with toluene, chlorobenzene, and diethyl ether (**b**) FWHM of MASnI_3_ films treated with toluene, chlorobenzene, and diethyl ether.

The influence of the antisolvent on the films’ phase structure and microstructure was studied by XRD. Figure 1 shows the X-ray diffraction patterns of the MASnI_3_ films treated with different antisolvents. The diffraction peaks that appeared at 14°, 28°, 31°, 38°, and 31° correspond to the (110), (220), (222), (224), and (314) peaks, respectively. These results are in good agreement with previous findings [15,16]; the FTO peaks are identified by the star symbol, The XRD results of the prepared MASnI_3_ films indicated good crystallinity. The intensity of the characteristic (110) peak increased enormously when toluene was used as an antisolvent compared to chlorobenzene and diethyl ether. The intensity decrement can be attributed to the excessive solubility of methylammonium iodide (MAI).

### Lattice Parameters

The lattice parameters were calculated using Equations (l) and (2) with two different theta diffraction angles:

(1) $$\frac{1}{d^{2}}=\frac{+k^{2}}{a^{2}}+\frac{l^{2}}{c^{2}}$$

(2) $$\eta \lambda =d_{hkl}\sin\left(\theta \right)$$

where *a*, *b*, *c* are the lattice parameters, *h*, *k*, *l* are Miller indices, *d_hkl_* is the interplanar distance, and *k* is the wavelength (0.154 nm).

The obtained lattice parameters of the main (110) peak were *a* = *b* = 8 Å, *c* = 11.9 Å, corresponding to the tetragonal structure.

Lattice Strain and Dislocation Density.

The lattice strain (ԑ) indicates a thin film’s crystal deformation calculated from Equation (3). The results obtained between 340 nm and 400 nm are summarized in Table 1.

(3) $$\beta \cos\left(\theta \right)=\frac{k\lambda }{D}+4ԑ\sin\left(\theta \right)$$

where *β* is the FWHM value, k is the dielectric constant, with the value k = 0.9, *λ* is the X-ray wavelength (*λ* is 0.1540 nm), *θ* is the Bragg diffraction angle, and *D* is the crystallite size (nm). Equation (4) below shows the estimated grain size dislocation density:

(4) $$\gamma =\frac{1}{D^{2}}$$

Table 1 shows that the three samples had different grain sizes, from 300 nm to 400 nm, when MASnI_3_ was treated with different antisolvents. The larger grain size of 400 nm was obtained with toluene. The lower effective lattice around 0.37 is explained by less deficiencies and distortions of the grains.

**Figure 2:**
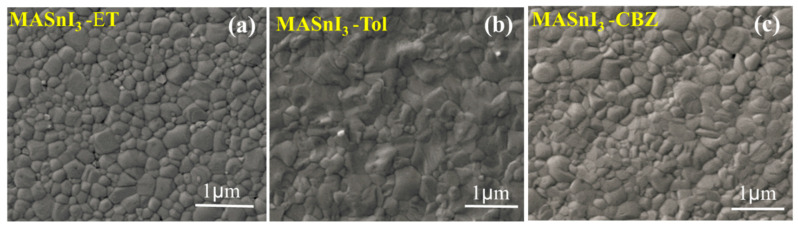
SEM images of MASnI_3_ films treated with (**a**) diethyl ether, (**b**) toluene, (**c**) chlorobenzene.

Figure 2 shows SEM images of MASnI_3_ treated with different antisolvents. The antisolvent quenching techniques presented here improved the crystalline quality of MASnI_3_ thin films and extended their lifetime. The antisolvent, in our case a perovskite solution, could not dissolve its components. Local regions of supersaturation are created when an antisolvent is applied, accelerating heterogeneous nucleation. High-quality films are obtained by optimizing the nucleation rate and the crystal growth rate. Generally, the antisolvent concentrations are kept high, on the basis of the solubility curve, and under the metastable limit on the seeding zone. These concentrations allow the growth of existing nuclei, or “seeds”, without creating new ones. The surface morphology of MASnI_3_ was examined with SEM. It was found that with diethyl ether, crystals poorly formed. Even chlorobenzene did not change significantly the morphology of the film. However, toluene significantly altered the films’ morphology, leading to the largest crystal size. These observations indicated that the morphology of MASnI_3_ changed in relation to the type of antisolvent [17,18]. The addition of antisolvent also produced fine needles that agglomerated easily. This proved that supersaturation plays a vital role in a crystallization system and influences the crystals’ size, shape, and degree of accumulation [19,20]. The remaining solvent is removed under 250 °C during the annealing treatment. The conversion of precursors in the perovskite phase was almost perfect in all samples, but the goal was to affect the surface.

**Figure 3:**
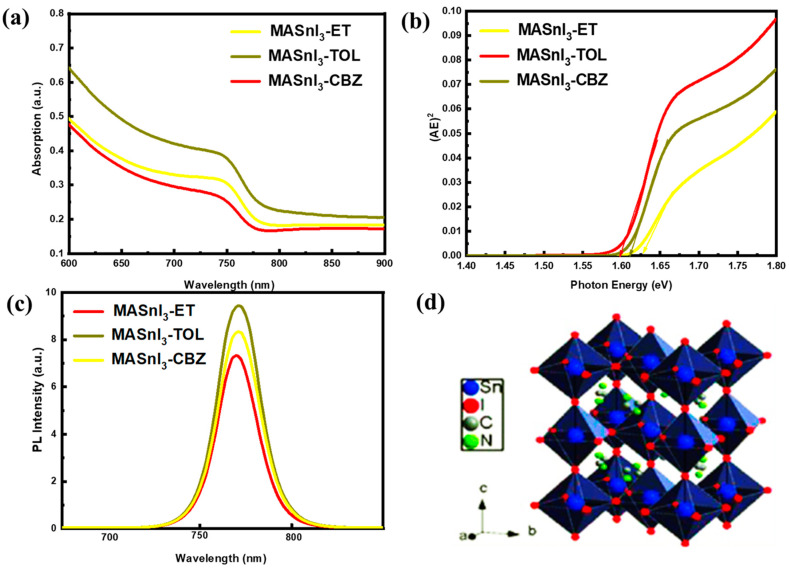
(**a**) Absorption of MASnI_3_ films, (**b**) bandgap, and (**c**) PL spectra after treatment with toluene, diethyl ether, and chlorobenzene, (**d**) structure of MASnI_3_.

All samples displayed a time-dependent PL spectrum under illumination and a high energy peak, as shown in Figure 3. Furthermore, storing a sample in dark conditions would “reset” the spectrum. Other authors made similar observations, and the “Hoke Effect” was also observed [21]. Figure 3 shows the characteristic PL peak of the three MASnI_3_ samples treated with different antisolvents, i.e., toluene, chlorobenzene, and diethyl ether, applied in the wavelength range from 750 to 800 nm [22]. The PL peak of the MASnI_3_ sample treated with toluene showed a higher intensity than that of the MASnI_3_ sample treated with diethyl ether or chlorobenzene. The MASnI_3_ sample treated with toluene also showed an optimal bandgap around 1.58 eV, calculated from the PL spectrum; the band gap calculated from the UV–visible spectrum was 1.6 eV, and the Stokes shift of MASnI_3_ treated with toluene showed a low value (Table 2). This can be related to phase transitions commonly found for halide perovskite [23,24,25].

## 4. Degradation Study

Oxygen and humidity significantly impact the stability of perovskite solar cells. Once the film is exposed to extreme environmental conditions, its degradation is due to the reduction to SnI_2_, MAI, and HI [26,27,28,29].

The stability of MASnI_3_ films was examined after four weeks from the treatment with the three antisolvents. To investigate the degree of degradation of the MASnI_3_ films, both fresh and four-week-old MASnI_3_ samples were analyzed by XRD and SEM; the MASnI_3_ aged samples were kept under 60% humidity in a dark environment.

**Figure 4:**
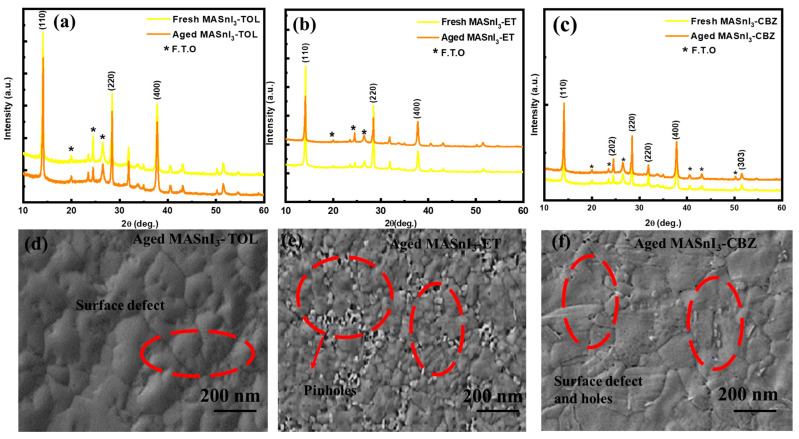
Degradation study of MASnI_3_ films treated with different antisolvents. (**a**) XRD pattern of fresh and four-week-old MASnI_3_ films treated with toluene, (**b**) XRD pattern of fresh and four-week-old MASnI_3_ films treated with diethyl ether, (**c**) XRD pattern of fresh and aged MASnI_3_ films treated with chlorobenzene, (**d**) SEM image of an aged MASnI_3_ film treated with toluene, (**e**) SEM image of a four-week-old MASnI_3_ film treated with diethyl ether (**f**) SEM image of a four-week-old MASnI_3_ film treated with chlorobenzene.

The XRD patterns for the four-week-old MASnI_3_ films treated with chlorobenzene and toluene antisolvents showed a slightly reduced intensity of the characteristic peaks (110) and (220) when compared to those of MASnI_3_ films treated with diethyl ether, which displayed a dramatic increase in the intensity of these peaks (Figure 4a–c). In this regard, chlorobenzene and toluene as antisolvents enhanced the stability of the MASnI_3_ films more than diethyl ether. The SEM images also supported this finding (Figure 4d–f). The surface morphology changes are observed in the SEM images of aged MASnI_3_ films with new grain boundaries and new defects. Therefore, the degradation process differs between the antisolvent-treated films.

## 5. Device Performance

We observed that the film thickness varied when different antisolvents were used, and similarly, the bandgap varied when MASnI_3_ was used. PSCs are more sensitive to variation in thickness related to the bandgap of their absorbing layers. As part of the simulations, we modeled the proposed solar cell in order to further analyze the impact of different antisolvents on its performance. In “Gold/Spiro-OMeTAD/MASnI_3_/Compact-TiO_2_/FTO/Glass”, MASnI_3_ was used as an absorber, Spiro-OmeTAD as a hole transport layer (HTL), Compact-TiO_2_ (C-TiO_2_) as an electron transfer layer (ETL), without taking into consideration mesoporous-TiO_2_ in the simulation model, and FTO was used as a substrate.

**Figure 5:**
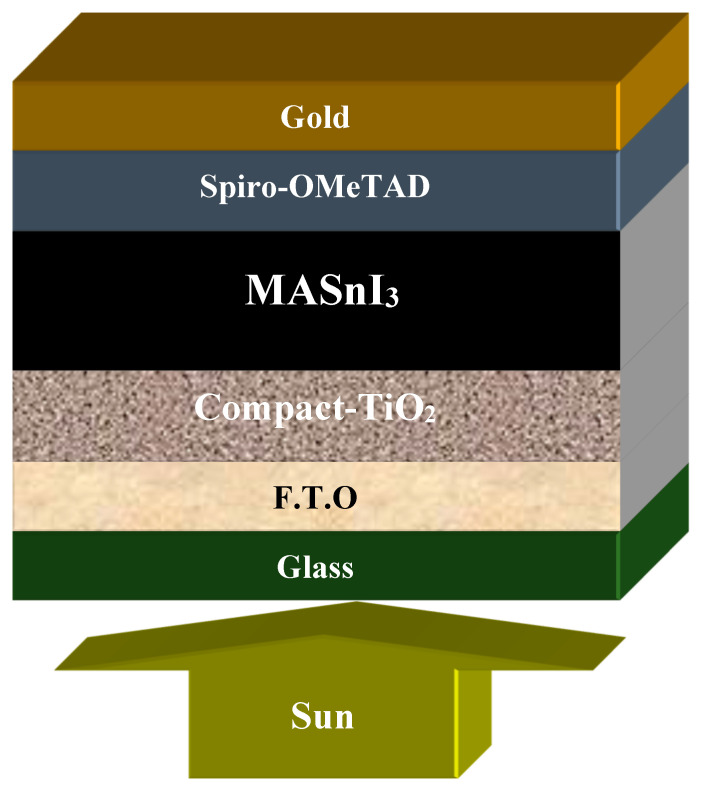
Schematic of a MASnI_3_-based solar cell structure.

Supplementary Table S1 and Table 3 provide a list of the simulation parameters used in SCAPS-1D software. Table 1 shows our experimental calculations, which we used in Table 2. The work function for back and front contact was used as default in SCAPS-1D. The proposed solar cell structure used in SCAPS-1D was arranged as shown in Figure 5. The layers of the cell are depicted in Figure 5.

The SCAPS-1D software version 3.3.10 was run under 1000 W/m^2^ illumination and at ambient temperature (300 K). The series resistance and shunt resistance were kept negligible and infinite, respectively, which are ideal values that experimentally do not exist up to these limits. The bandgap and thickness of MASnI_3_ are shown in Table 3.

**Figure 6:**
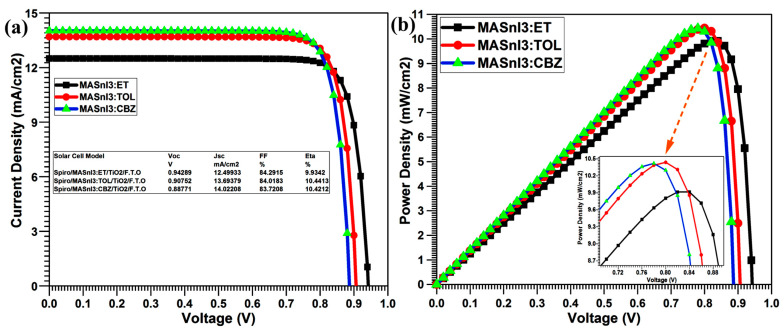
(**a**) J–V and (**b**) P–V characteristics curves of MASnI_3_-based solar cell.

Figure 6a,b illustrate the J–V and P–V curves for MASnI_3_, respectively, and demonstrate how the different antisolvents affected the results. Toluene had a positive impact on the device; the P–V angles delivered the highest power when using toluene as an antisolvent.

Diethyl ether antisolvent-based MASnI_3_ produced V_oc_ of 0.94 V, J_sc_ of 12.49 mA/cm^2^, FF of 84.29%, and Eta of 9.93% during the simulation of solar cells. We notice that diethyl ether is less effective because, chlorobenzene V_oc_, J_sc_, FF, and Eta were registered as 0.88 V, 14.02 mA/cm^2^, 83.72%, and 10.42%, respectively, which was good in comparison to when ET was used as antisolvent as shown in Table 4.

In summary, Toluene is the most profitable antisolvent because it gave excellent results compared to the other two antisolvents. With Toluene treatment for MASnI_3_ films, we record V_oc_, J_sc_, FF, and Eta as 0.90 V, 13.69 mA/cm^2^, 84.01%, and 10.44%, respectively.

**Figure 7:**
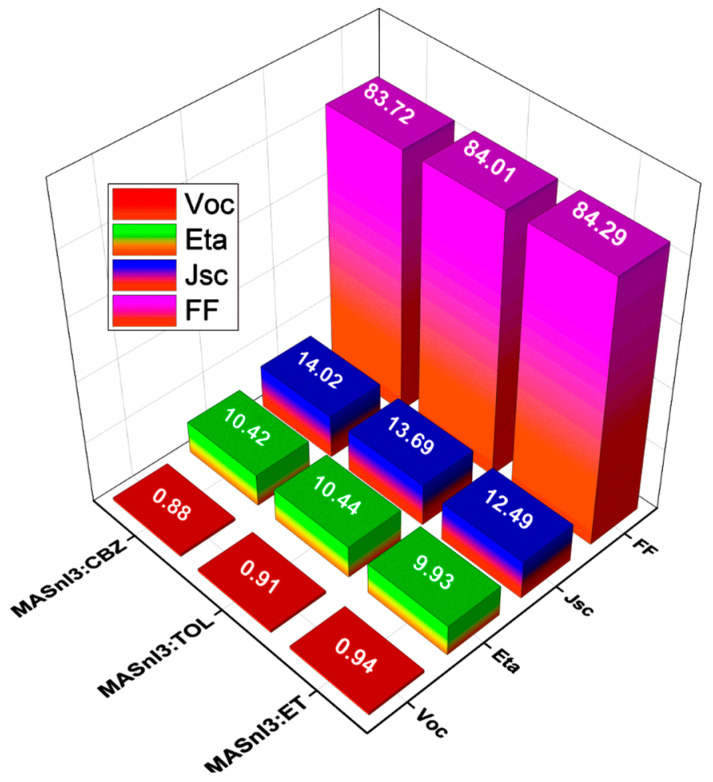
Characteristic parameters of a MASnI_3_-based solar cell when using the different antisolvents.

Here, in Figure 7, we compare the characteristic parameters of MASnI_3_ solar cells based on different antisolvents used in the solution. We can observe that toluene would be the optimal antisolvent for preparing the MASnI_3_ absorber layer as it gives us the best results of the characteristic parameters for the solar device.

## 6. Manufacture of the Spiro-OMeTAD/MASnI_3_/C-TiO_2_/FTO Device

**Figure 8:**
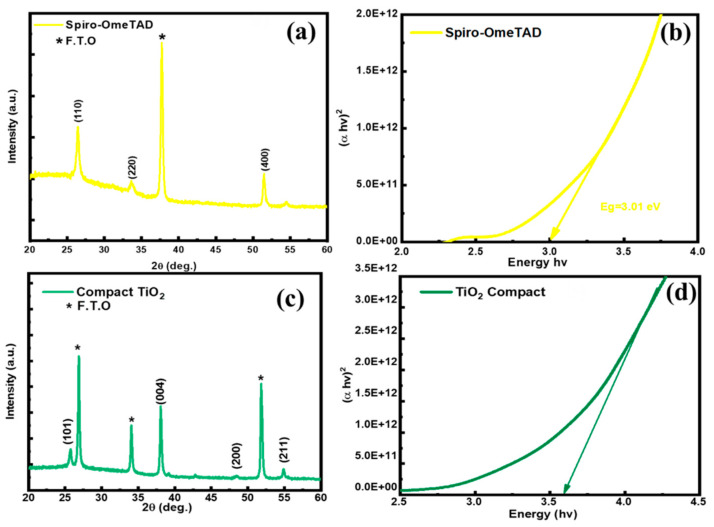
(**a**) XRD Pattern of the Spiro-OmeTAD film, (**b**) bandgap spectra of the Spiro-OmeTAD film, (**c**) XRD pattern of the TiO_2_ compact layer, (**d**) bandgap spectra of the TiO_2_ compact layer. “3.5E+12” represents “3.5 × 10^12^”, same applies to other E notations in the figure.

We report the XRD analysis for the Spiro-OMeTAD film with characteristic peaks at (110), (220), and (400) (Figure 8a). These results are in agreement with previous studies [30]. When calculating the experimental bandgap after absorbance characterization, we obtained 3.0 eV, which is the optimal bandgap, also previously reported [31]. For compact TiO_2_, the XRD spectrum showed characteristic peaks at (101), (004), (200,) and (211) (Figure 8c). We found the same characteristic peaks when comparing these XRD spectrum with those in the literature [32]. The absorption analysis for TiO_2_ revealed an experimental bandgap of 3.6 eV (Figure 8d).

**Figure 9:**
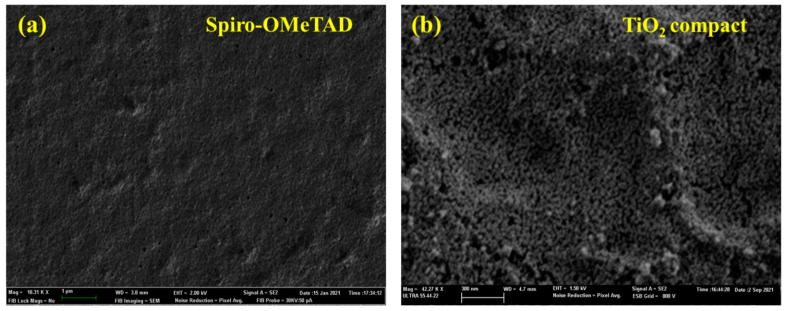
(**a**) SEM image of the Spiro-OMeTAD film, (**b**) SEM image of the TiO_2_ compact layer.

Figure 9 shows SEM images of both the Spiro-OMeTAD and the TiO_2_ layers. The SEM image of the TiO_2_ films showed a smooth and homogenous surface. The Spiro-OmeTAD film showed a regular surface with no holes.

The device Spiro-OMeTAD/MASnI_3_/C-TiO_2_/FTO was fabricated. The thickness of TiO_2_ was approximately 200 nm, that of the MASnI_3_ film was between 300 and 400 nm, and that of the Spiro-OMeTAD layer was around 100 nm. The gold layer had a thickness of around 50 nm.

The results of four planar devices named Sx (x = (1, 2, 3, 4)) containing four separated pixels are presented. Each showed high efficiency, resulting in 30 solar cells manufactured simultaneously under similar conditions. Pixel number S x_1 refers to the first pixel in cell Sx. The cells were concealed in a metal aperture with an area of 0.1cm^2^.

A solar simulator was used to measure the device efficiencies and curves within 24 h from the thermal evaporation of gold on the surface to obtain a back electrode. The S_1_ device showed a PCE average of 9.44% in the case of toluene treatment, under 1 Sun AM 1.5 illumination using the forward scan mode, with a scale setting of −0.2 V to short circuit 1.2 V, a sampling rate of 10 mV/s; we did not notice any hysteresis. The average performance parameters are listed in Table 5.

We then fabricated Gold/Spiro-OMeTAD/MASnI_3_/Compact-TiO_2_/FTO/Glass devices with MASnI_3_ films treated with toluene or chlorobenzene as antisolvents. We did not fabricate devices using MASnI_3_ films treated with diethyl ether because this antisolvent showed worse stability and morphology.

**Figure 10:**
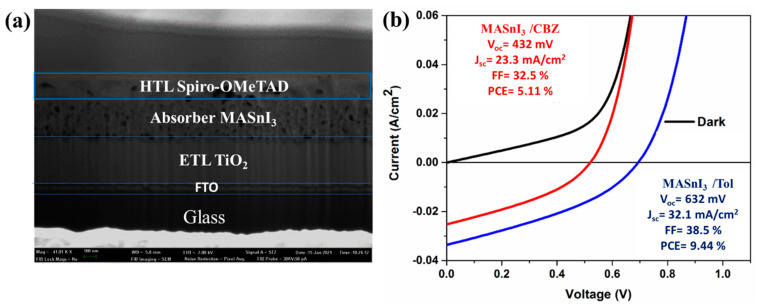
(**a**) Cross section of a lead-free perovskite solar cell. (**b**) I–V performance of perovskite MASnI_3_ (black color = dark curves are identical for both devices, Red color = chlorobenzene, Blue color = toluene) (**b**) cross-section of a lead-free perovskite solar cell.

Figure 10 illustrates the photovoltaic performance of MASnI_3_. The J–V curves showed that the prepared PSC realized a remarkable PCE improvement of 5.11% when using MASnI_3_–chlorobenzene, an open-circuit voltage (V_oc_) of 521.8 mV, a short-circuit current (J_sc_) of 25.6 mA cm^−2^, and a fill factor (FF) of 34.59%. PCE improvement was of 9.44% after treatment with toluene, using V_oc_ of 694.6 mV, J_sc_ of 32.1 mA cm^−2^, and an FF of 38.09%, which was significantly higher than that measured for the MASnI_3_–chlorobenzene-based solar cell. It is possible that the use of toluene increased the quality and stability of the film by increasing the carrier concentration and decreasing the electron–hole recombination, thus enhancing the photoconversion efficiency from 5.11% to 9.44%. A high value observed for J_sc_ is normally due to the exciting electrons generated following light absorption; the higher the light absorption, the higher the electron excitation, and as a resultant, J_SC_ will be high too [33,34]. Some results were not easy to reproduce because of the low-cost techniques used for the manufacture of the devices and because of variable temperature and humidity conditions in addition [35,36], some interface defects were noticed. Despite this, the results were generally quite reproducible with small differences. The obtained results might contribute to the production and commercialization of stable and efficient photovoltaic devices.

## 7. Conclusions

In this work, MASnI_3_ thin films were successfully prepared by the one-step spin coating technique. The effect of different antisolvents, i.e., diethyl ether, chlorobenzene, and toluene, on films’ properties was investigated using XRD, SEM, PL, and optical analyses.

The XRD analysis revealed that all samples had an extraordinary (110) peak intensity after treatment of the MASnI_3_ film. The XRD results showed that toluene led to superior crystallinity, reflected by the intensity of the (110) peak. This result was supported by SEM results, that showed that toluene led to increased grain size. Furthermore, the sample obtained with toluene treatment showed the highest absorbance. Our results suggest that using toluene to carry out the antisolvent quenching step in a one-step spin coating will lead to superior perovskite films and higher efficiencies of related devices. Using SCAPS-1D software, we also determined the effect of the three different antisolvents, i.e., diethyl ether, chlorobenzene, and toluene, on solar cell performance, obtaining the efficiencies of 9.93, 10.42, and 10.44%, respectively. We therefore conclude that toluene is the optimal antisolvent for the MASnI_3_ absorber layer. Additionally, we produced the device FTO/TiO_2_/MASnI_3_/Spiro-OMeTAD/Au, which showed a remarkable PCE of 9.11% when using a MASnI_3_ film treated with toluene.

## Figures and Tables

**Table 1:** Parameters of XRD spectra of MASnI_3_ films treated with toluene, chlorobenzene, and diethyl ether.

Sample ID	Grain Size (nm)	Dislocation Density (nm^−1^)	Lattice Strain (ԑ)
MASnI_3_-ET	340	1.05 × 10^−5^	0.38
MASnI_3_-CBZ	360	0.90 × 10^−5^	0.39
MASnI_3_-Tol	400	0.62 × 10^−5^	0.37

**Table 2:** Optical bandgaps of the MASnI_3_ samples treated with toluene, diethyl ether, and chlorobenzene.

Sample	λ (nm)	Eg from PL (eV)	λ (nm)	Eg from UV (eV)	Stokes Shift (meV)
MASnI_3_-ET	779	1.59	750	1.63	40
MASnI_3_-TOL	782	1.58	759	1.60	20
MASnI_3_-CBZ	787	1.57	760	1.62	50

**Table 3:** Bandgap variation for MASnI_3_ calculated from the UV–visible spectra, using diethyl ether, toluene, and chlorobenzene.

Sample	Thickness (nm)	Band Gap-UV (eV)
MASnI_3_-ET	200	1.63
MASnI_3_-TOL	210	1.60
MASnI_3_-CBZ	210	1.62

**Table 4:** Experimental Characteristics Parameters MASnI_3_-based solar cell.

Solar Cell Model	V_oc_	J_sc_	FF	Eta
	V	mA/cm^2^	%	%
Spiro-OMeTAD/MASnI_3_: ET/TiO_2_/FTO	0.94	12.49	84.29	9.93
Spiro-OMeTAD/MASnI_3_: TOL/TiO_2_/FTO	0.90	13.69	84.01	10.44
Spiro-OMeTAD/MASnI_3_: CBZ/TiO_2_/FTO	0.88	14.02	83.72	10.42

**Table 5:** Average performance of the Spiro-OMeTAD/MASnI_3_/C-TiO_2_/FTO devices.

Device. ID	Size	η	FF	$V_{oc}$	$J_{sc}$
	[cm^2^]	[%]	[%]	[V]	[mA/cm^2^]
S_1_-TOL	0.1	9.44	38.09	0.69	32.01
S_2_-TOL	0.1	8.31	37.07	0.60	29.21
S_3_-CBZ	0.1	5.11	34.59	0.52	25.60
S_4_-CBZ	0.1	4.26	33.62	0.51	24.25

## Data Availability

All data is included in the manuscript.

## Supplementary Materials

The following supporting information can be downloaded at: https://www.mdpi.com/article/10.3390/nano12172901/s1, Table S1: Simulation Parameters of MASnI_3_-based solar cell.
