# Supplementary material for: Manufacture of High-Efficiency and Stable Lead-Free Solar Cells through Antisolvent Quenching Engineering
Source: Nanomaterials (Basel). 2022 Aug 23;12(17):2901. doi: 10.3390/nano12172901 (PMC9457650; doi:10.3390/nano12172901)
Supplement: Supplementary file 1 [file nanomaterials-12-02901-s001.zip › nanomaterials-1847047-supplementary.pdf]

Supplementary Materials

Table S1. Simulation Parameters of $MASnI_3$-based solar cell.

Parameters	Spiro	$MASnI_3$	TiO_2	FTO
**Thickness (*um*)**	0.20	**Variable**	0.05	0.5
**Bandgap (eV)**	3.17	**Variable**	3.2	3.5
**Electron affinity (eV)**	2.05	3.49	3.9	4.0
**Dielectric Permittivity**	3.00	20.0	9.0	9.0
**CB Effective Density of States ($1/cm^3$)**	2.2×10^{18}	1.0E+19	2.0E+17	2.2E+18
**VB Effective Density of States ($1/cm^3$)**	1.8×10^{19}	1.0E+19	6.0E+17	1.8E+19
**Electron Thermal Velocity (cm/s)**	1.0E+7	1.0E+7	1.0E+7	1.0E+7
**Hole Thermal Velocity (cm/s)**	1.0E+7	1.0E+7	1.0E+7	1.0E+7
**Electron Mobility (cm^2/Vs)**	2.0E-4	2.30E+3	1.00E+2	2.00E+1
**Hole Mobility (cm^2/Vs)**	2.0E-4	3.20E+2	2.50E+1	1.00E+1
**Donor Density ND ($1/cm^3$)**	0.0E+0	1.0E+9	1.0E+17	1.0E+17
**Acceptor Density NA ($1/cm^3$)**	2.0E+19	1.0E+9	0.0E+0	0.0E+0
